# Induction of cytotoxicity of *Pelagia noctiluca *venom causes reactive oxygen species generation, lipid peroxydation induction and DNA damage in human colon cancer cells

**DOI:** 10.1186/1476-511X-10-232

**Published:** 2011-12-09

**Authors:** Yosra Ayed, Manel Boussabbeh, Wiem Zakhama, Chayma Bouaziz, Salwa Abid, Hassen Bacha

**Affiliations:** 1 Laboratory for Research on Biologically Compatible Compounds, Faculty of Dentistry, Rue Avicenne, 5019 Monastir, Tunisia; 2 University of Jendouba, Cité AlFaeiz rue Jamil Boutheina Jendouba 8100, Tunisia

**Keywords:** *Pelagia noctiluca*, Jellyfish, Venom, Cytotoxicity, Oxidative stress, DNA fragmentation

## Abstract

**Background:**

The long-lasting and abundant blooming of *Pelagia noctiluca *in Tunisian coastal waters compromises both touristic and fishing activities and causes substantial economic losses. Determining their molecular mode of action is, important in order to limit or prevent the subsequent damages. Thus, the aim of the present study was to investigate the propensity of *Pelagia noctiluca *venom to cause oxidative damage in HCT 116 cells and its associated genotoxic effects.

**Results:**

Our results indicated an overproduction of ROS, an induction of catalase activity and an increase of MDA generation. We looked for DNA fragmentation by means of the comet assay. Results indicated that venom of *Pelagia noctiluca *induced DNA fragmentation. SDS-PAGE analysis of *Pelagia noctiluca *venom revealed at least 15 protein bands of molecular weights ranging from 4 to 120 kDa.

**Conclusion:**

Oxidative damage may be an initiating event and contributes, in part, to the mechanism of toxicity of *Pelagia noctiluca *venom.

## Background

*Pelagia noctiluca *[1] (family Pelagiidae, Semaestomeae, Scyphozoa) is a highly venomous jellyfish species [2], widely distributed in different parts of the Mediterranean Sea [2,3] and in the Atlantic Ocean [5]. *Pelagia noctiluca *(*P. noctulica*) has not caused human fatalities, but in spite of this, it can have profound ecological and socio-economic consequences when it appears in huge numbers during outbreaks [2-4].

As a matter of fact, events of massive occurrence of *P. noctulica *can be detrimental to aquaculture causing mortality of caged fish [6], to tourism by curtailing bathing activities [7], as for fishing activities, since in several cases, it was impossible to separate the biomass of medusae from fishes [8].

One of the most distinctive aspects of jellyfish physiology is related to its biologically active components and organelles contained in specialized cells called nematocysts. They are located along the tentacles and body. These organelles contain toxins and discharge their content upon an appropriate stimulation [9]. The venom of *P. noctulica *is of protein nature and contains peptides. It is antigenic and possesses dermonecrotic and hemolytic properties [10]; Cytolytic and neurotoxic effects have also been shown by several biological assays. The haemolytic effect is the most well studied activities of the venom [11-14]. Electrophoretical analyses recognized eight different fractions, distinguished by molecular mass [15].

The protein nature of venom [16] was further confirmed by Mariottini et al. [17]. *P. noctiluca *venom also caused an increase of ATP levels in treated cells followed by a moderate decrease [17]. This is an atypic response since most toxicity studies reported decrease of ATP levels in stress-exposed cells [18,19] and organisms [20]. The toxicological nature of this venom has neither been characterized nor clearly described yet. Understanding the molecular mode of action of the cytosolic venom of *P. noctulica *is essential to predict their harmful effects on human health.

In this regard, our study aimed to evaluate the level of toxicity of *P. noctiluca *venom on HCT116 cells. We looked for the effects of the venom of *P. noctiluca *on cell viability, oxidative status and DNA fragmentation. To characterize the proteinous components of *P. noctiluca *venom, we separated the venom proteins using SDS-PAGE.

## Materials and methods

### 1. Chemicals

3-4, 5-dimethylthiazol-2-yl, 2, 5-diphenyltetrazolium bromide (MTT), Cell culture medium (RPMI1640), foetal calf serum (FCS), phosphate buffer saline (PBS), trypsin-EDTA, penicillin and streptomycin mixture and l-glutamine (200 mM) were from GIBCO-BCL (UK). 2, 7-Dichlorofluoresce diacetate (DCFH-DA) was supplied by Molecular Probes (Cergy Pontoise, France). Low melting point agarose (LMA) and normal melting point agarose (NMA) were purchased from Sigma (St. Louis, MO). All other chemicals used were of analytical grade.

### 2. Preparation of nematocysts

The nematocysts isolation method has been previously described by Arillo et al. [21]. Briefly, specimens of *P. noctiluca *were collected in the Strait of Monastir. The oral arms were excised and submerged in distilled water for 5 h at 4°C. The ratio of organic tissue to distilled water was approximately 1:5. After a complete detachment of the epidermis the tissue was removed from the suspension containing both epidermis and undischarged nematocysts deriving from the osmotic rupture of nematocysts. The nematocysts, still attached to the epidermal tissue, were separated by stirring. The nematocysts suspension was repeatedly washed in distilled water and filtered through plankton nets (40, 60 and 100 μm mesh, respectively) to remove most of the tissue debris, and then centrifuged at 4°C (ALC PK 120R, 4000 g for 5 min). The content, purity and integrity of nematocysts (cnidocysts) were controlled microscopically, and the nematocysts concentrate was stored at -80°C until further use [22].

### 3. Nematocysts lysis and protein extraction

Crude venom was extracted by sonication on ice (Sonoplus, 70 mHz, 30 times, 20 s) of nematocysts as described by Marino et al. [22]. After sonication, the suspension was centrifuged at 11, 000 rpm for 5 min at 4°C. The supernatant was carefully removed, filtered and lyophilized.

### 4. Protein determination

The protein content was determined according to the Bradford method (BioRad Labs, Hercules, CA) [23]. In the following, all mention of ''venom concentration'' refers to protein concentration expressed in units of μg ml^-1^.

### 5. Cell culture and treatment

The human colon cancer cell line HCT 116 (wt) was gifted by Professor Olivier Micheau (Faculty of Medicine and Pharmacy, Univ. Bourgogne, Dijon). HCT 116 (wt) cells were grown as monolayer culture in RPMI 1640 medium (pH 7-8) supplemented with 10% fœtal calf serum (FCS), 1% L-glutamine (200 mM), 1% of mixture Penicillin (100 IU/ml) and Streptomycin (100 μg/ml) incubated at 37°C in an atmosphere of 5% CO2.

### 6. Determination of cell viability

Cytotoxicity of pelagia crude venom was defined using the colorimetric method described by Carmichael et al. [24]. The MTT test assesses cell metabolism based on the ability of the mitochondrial succinate-dehydrogenase to convert the yellow compound MTT to a blue formazan dye. The amount of dye produced is proportional to the number of live metabolically active cells.

Cells were seeded on 96-well culture plates (Polylabo, France) at 10^5 ^cells/well and treated with increasing concentrations of crude venom extract at 37°C. After 24 h, the culture medium was replaced by 200 μl medium containing 0.5 mg/ml MTT and the plates were incubated 3 hours at 37°C. The medium was then removed and replaced by 200 μl of (0, 04 M HCl/isopropanol) to solubilize the converted purple dye in culture plates. The absorbance was measured on a spectrophotometer microplate reader (Dynatech 4000) at 545 nm.

Cell viability was expressed as the relative formazan formation in treated samples as compared to control cells (untreated cells) [(A545 treated cells/A545 control cells) 100%]. The inhibitory concentration of 50% of cell viability (IC50) values defined as the concentration inducing 50% loss of cell viability.

### 7. Measurement of reactive oxygen species (ROS) production by DCFH-DA method

The intracellular amounts of ROS were measured by a fluorometric assay with 2', 7'-dichlorofluorescein diacetate (DCFH-DA) used extensively to monitor oxidation in biological systems as a well established compound to detect and quantify intracellular produced such as superoxide radical, hydroxyl radical, and hydrogen peroxide [26,27]. The conversion of the non-fluorescent (DCFH-DA) to the highly fluorescent 2', 7'- dichlorofluorescein product (DCF) (λ max = 522 nm) happens in many steps. The fluorescent probe, after diffusing in the cell membrane, is hydrolysed by intracellular esterases to non-fluorescent dichlorofluorescein (DCFH), which is trapped inside the cells then oxidized to fluorescent DCF through the action of peroxides in presence of ROS [28].

At 50% confluence, HCT 116 cells were incubated with different concentration of P. noctiluca venom (80, 160, 320, and 640 μg/ml) at a treatment time of 24 h, a positive control was treated with 75 μM of H2O2 during 5 min. Negative control corresponds to untreated cells. They were then treated with 20 μM DCFH-DA. Intracellular production of ROS was measured after 30 min incubation at 37°C by fluorometric detection of DCF oxidation on a fluorimeter (Biotek FL × 800) with an excitation wavelength of 485 nm and emission wavelength of 522 nm. The DCF fluorescence intensity is proportional to the amount of ROS formed intracellularly. Results are expressed as the ratio DCF- induced *P. noctiluca *venom fluorescence/DCF-induced control fluorescence.

### 8. Determination of Catalase activity

Catalase activity was measured in HCT116 cells extracts spectrophotometrically at 240 nm, 25°C according to Clairbone [29]. HCT 116 cells were seeded on 6-well culture plates (Polylabo, France) at 75 × 10^4 ^cells/well for 24 h of incubation. After, the cells were incubated with *P. noctiluca *crude venom at 80, 160, 320, and 640 μg/ml, for 24 h at 37°C. Briefly, 20 μl of the cell extracts were added in the quartz cuvette contain 780 μl phosphate buffer and 200 μl of H2O2 0.5 M. The activity of catalase was calculated using the molar extinction coefficient (0.04 mM-1 cm-1). The results were expressed as μmol of H2O2/min/mg of proteins.

### 9. Lipid peroxidation

Lipid peroxidation was assayed by the measurement of malondialdehyde (MDA) according to the method of Ohkawa et al. [30]. The cells were seeded in 6-well plates at 6 × 10^5 ^cells/well. After 24 h of incubation, they were exposed to different concentrations of *P. noctiluca *venom extract (80, 160, 320, and 640 μg/ml) corresponding to IC50/4, IC50/2, IC50, and 2 × IC50 s of crude venom in HCT 116 cells for 24 h, followed by 1 mM H2O2 for 2 h. The cells were then washed with cold PBS, scraped and lysed by homogenization in ice-cold 1.15% KCl. Samples containing 100 ml of cell lysates were combined with 0.2 ml of 8.1% SDS, 1.5 ml of 20% acetic acid adjusted to pH 3.5 and 1.5 ml of 0.8% thiobarbituric acid. The mixture was brought to a final volume of 4 ml with distilled water and heated to 95°C for 120 min. After cooling to room temperature, 5 ml of mixture of n- butanol and pyridine (15:1, v:v) was added to each sample and the mixture was shaken vigorously. After centrifugation at 15, 000 rpm for 10 min, the supernatant fraction was isolated and the absorbance measured at 532 nm. The concentration of MDA was determined according to a standard curve.

### 10. DNA damage assessed by the comet assay

Comet assay, also known as single cell gel electrophoresis (SCGE), is a visual and sensitive technique for measuring DNA breakage in individual mammalian cells. HCT 116 cells were seeded on 6-well culture plates Polylabo, France) at 75 × 10^4 ^cells/well for 24 h of incubation and were re-incubated as described above in the presence of *P. noctiluca *crude venom at 80, 160, 320, and 640 μg/ml for 24 h at 37°C. H2O2 (75 μM) was used as a positive control. Approximately 2 × 10^4 ^cells were mixed with 1% low melting point agarose (LMP) in PBS and spread on a microscope slide previously covered with a 1% normal melting agarose (NMP) in PBS layer. After agarose solidification, cells were treated with an alkaline lysis buffer (2.5 M NaCl, 0.1 M EDTA, 10 mM Tris, pH 10, 1%(v/v) Triton X-100 and 10% (v/v) DMSO)for 1 h at 4°C, then the DNA was allowed to unwind for 40 min in the electrophoresis buffer (0.3 M NaOH, 1 mM EDTA, pH > 13). The slides were then subjected to electrophoresis in the same buffer for 30 min at 25 V and 300 mA. Slides were then neutralized using a Tris buffer solution (0.4 M Tris, pH 7.5) for 15 min. After staining the slides with ethidium bromide (20 μg/ml), the comets were detected and scored using a fluorescence microscope. The experiment was done in triplicate. The damage is represented by an increase of DNA fragments that have migrated out of the cell nucleus during electrophoresis and formed an image of a "comet" tail. A total of 100 comets on each slide were visually scored according to the intensity of fluorescence in the tail and classified by one of five classes as described by Collins et al., [31]. The total score was evaluated according to the following equation: (% of cells in class 0 × 0) +(% of cells in class 1 × 1) +(% of cells in class 2 × 2) +(% of cells in class 3 × 3)+(% of cells in class 4 × 4).

### 11. SDS-PAGE

Protein species were observed by polyacrylamide gel electrophoresis (SDS-PAGE) as described previously [32]. Jellyfish venom protein (200 μg) were diluted (1:1) with sample buffer (50 mM Tris pH 6.8, 2% SDS, 20% glycerol, 2% 2- mercaptoethanol and 0.04% bromophenol blue) and were then boiled for 3 min. Running gels of 5% acrylamide and stacking gels of 12% acrylamide were used. The gels were stained with Coomassie R-250. The molecular size marker, 6-170 kDa (protein standards, Invitrogen, CA, USA), was run parallel with venom sample for molecular weight estimation

### 12. Statistical analysis

Each experiment was done three times separately. Values were presented as means ± S.D. Statistical differences between control and treated groups for all expressions were determined by Student's test. Differences were considered significant at P < 0.05.

## Results

### 1. Inhibition of cell proliferation

**Figure 1:**
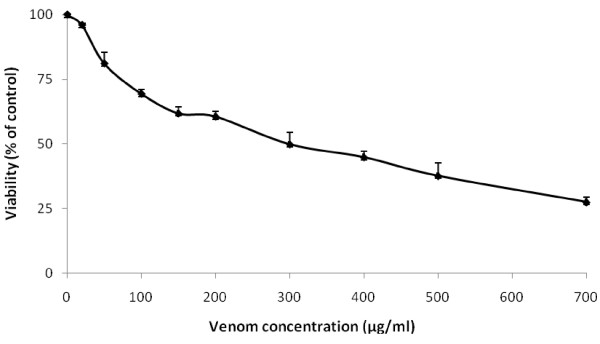
**Cytotoxic effect of *P. noctiluca *crude venom on HCT 116 cells**. Cells were treated with venom at the indicated concentrations for 24 h. Cell viability was determined using the MTT assay and expressed as percentages of control which was exposed to vehicle only. Control value was taken as 100%. Data are expressed as the mean ± S.D.

Cytotoxic effects of *P. noctiluca *crude venom on HCT 116 cells after 24 h incubation was measured by MTT assay. Results showed a dose-dependent inhibition of cell viability at increasing concentrations of *P. noctiluca *venom (Figure 1). The IC50 value as determined after 24 h of cell treatment from the viability curve was approximately about 320 μg/ml.

### 2. Oxidative status

#### 2. 1. Measurement of reactive oxygen species (ROS) production

**Figure 2:**
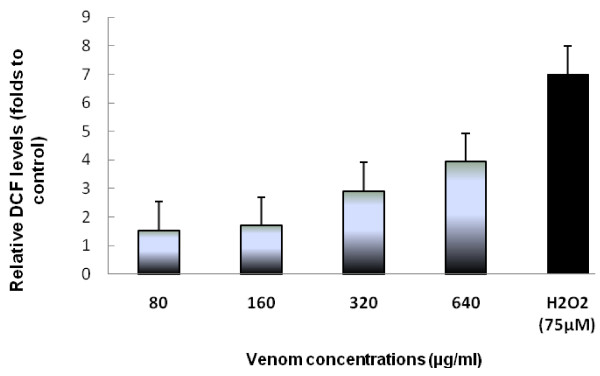
**Levels of relative fluorescent DCF production, after exposure of HCT 116 cells to 80, 160, 320, and 640 μg/ml corresponding to IC50/4, IC50/2, IC50, and 2 × IC50 s of crude venom of *P. noctiluca *for 24 h**. Fluorescent DCF is the result of DCFH oxidation by a variety of peroxides. H2O2, 75 μM was used as a positive control. This graph is a representative of three independent experiments. Data are expressed as the mean ± S.D.

To check the oxidative stress status in HCT 116 cells in response of crude venom of *P. noctiluca *at different concentrations (80, 160, 320, and 640 μg/ml) corresponding to IC50/4, IC50/2, IC50, and 2 × IC50 s of crude venom), we measured the production of fluorescent DCF (the result of DCFH oxidation by a variety of peroxides). Results shown in Figure 2, demonstrated that crude venom induced an increase of ROS generation in a concentration manner (Figure 2). In fact, ROS production exceed 1.5; 1.7; 3 and 4 fold to control for respectively IC50/4, IC50/2, IC50, and 2 × IC50 s of crude venom in HCT 116 cells after 24 h. Positive control treated with H2O2 (75 μM) which went beyond 7 fold to control (Figure 2).

#### 2. 2. Catalase activity

**Figure 3:**
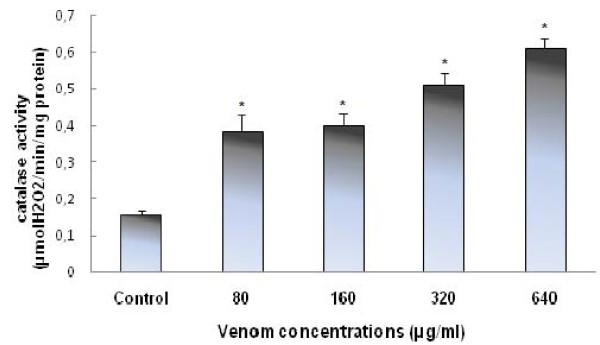
***P. noctiluca *crude venom induced catalase activity on HCT 116 cells after 24 h of treatment**. Results are expressed as mean ± S.D from three independent experiments. *Values are significantly different at p < 0.05 as compared to the control.

In order to investigate the responses of the reactive oxygen scavenging system of HCT 116 cells after 24 h of exposure to *P. noctiluca *venom the activity of catalase were measured. Results are illustrated in Figure 3. The catalase activity was found to be significantly increased in HCT 116 cells. It passed from 0.15 ± 0.08 in control cells (untreated cells) to 0.61 ± 0.095 μmol of H2O2 decomposed/min/mg of protein in treated cells with the highest concentration of Pelagia crude venom (640 μg/ml).

#### 2. 3. Induction of lipid peroxidation

**Figure 4:**
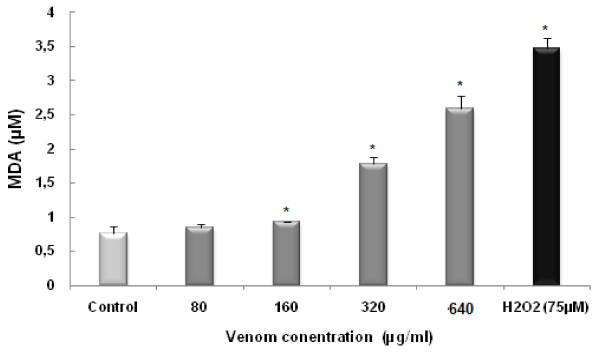
**Lipid peroxidation induced in HCT 116 cells, incubated for 24 h with venom of *P. noctiluca *at 80, 160, 320, and 640 μg/ml, measured by the production of malondialdehyde (MDA)**. Results were expressed as means ± S.D. from at least three independent experiments. * Values are significantly different at p < 0.05 as compared to the control.

*P. noctiluca *venom induced lipid peroxidation. Indeed, after 24 h incubation with different concentration of crude venom (80, 160, 320, and 640 μg/ml), the MDA level detected in HCT 116 cells increased from basal value of 0.78 ± 0.1 μM at 80 μg/ml of pelagia venom (p < 0.05) to 2.6 ± 0.18 μM (p < 0.05) at 640 μg/ml of crude venom (Figure 4).

### 3. Effect of the venom on DNA damage assessed by the comet assay

**Figure 5:**
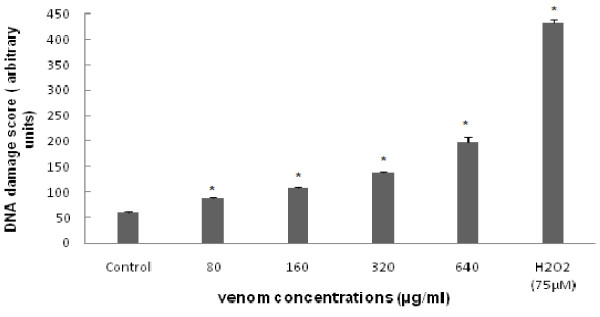
**Total DNA damage observed on HCT116 cells after treatment with *Pelagia *venom (80, 160, 320, and 640 μg/ml) for 24 h of treatment**. H2O2 (75 μM) was used as a positive control. DNA stand breaks were detected by the standard comet assay. * Values are significantly different at p < 0.05 as compared to the control.

DNA damage was analyzed using the Comet assay; a sensitive method for detecting DNA strand breaks in individual cells, and a versatile tool that is highly efficacious in human bio-monitoring of natural or environmental compounds [33]. The high sensitivity of the Comet assay, and the provided ability to measure DNA damage in individual cells, has destined it to become a tool in rapidly predicting genotoxicity of compounds of interest. The induction of DNA damage in HCT 116 cells after exposure to crude venom of *P. noctiluca *for 24 h was studied. The amount of DNA damage reaches about 3 folds to the control value after 24 h of crude venom at the highest concentration. Indeed, as shown in Figure 5, crude venom extract at concentrations of IC50/4, IC50/2, IC50, and 2 × IC50, induced 87.6 ± 2.08, 107.6 ± 3.51, 137.6 ± 4.04 and 198.66 ± 9.5 of total score of DNA damage as compared to 59.6 ± 2.08 of the score in control cells (untreated cells). H_2_O_2 _(75 μM) treated cells (positive control) formed a clear comet of more than 7 folds of untreated control cells (Figure 5).

### 4. SDS-PAGE of *P. noctiluca *Venom

**Figure 6:**
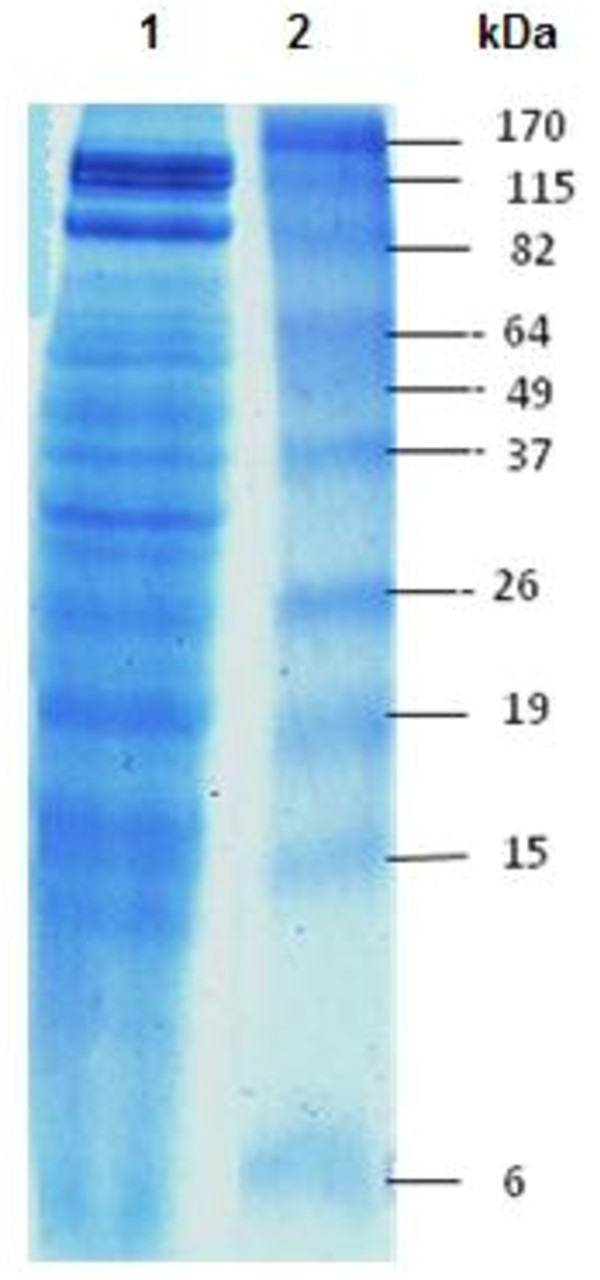
**SDS-PAGE separation of *P. noctiluca *venom proteins**. Electrophoresis was carried out according to Laemmli [32] using 12% polyacrylamide gel. Jellyfish venom protein (200 μg) was electrophoresed for 90 min at 100 V constant current in room temperature, using Tris-glycine running buffer. The molecular weight size marker (lane 2), in the range of 6 -170 kDa, was run parallel with venom sample (lane 1) for molecular weight estimation. Protein bands were visualized by staining gels with Coomassie R-250 dye.

Electrophoretical analysis of *P. noctiluca *Venom revealed a number of bands of varying size (Figure 6). 15 bands appeared after staining of the SDS-PAGE gel. The molecular mass of these bands was 120; 115; 80; 70; 66; 64; 55; 45; 37; 33; 29; 20; 16; 14; 4 kDa, respectively. The components of jellyfish venom were complex.

## Discussion

Oxidative stress is a term commonly used to denote the imbalance between the concentrations of reactive oxygen species (ROS) and the antioxidative defense mechanisms of the body. Compelling evidences suggest that an excess of ROS production to such an extent that cellular defences are overwhelmed and the cell is injured, is largely considered as playing a key role in a variety of human diseases [34,35]. To counteract the detrimental effects of oxidative stress, cells deploy antioxidant defenses, activate damage removal and repair systems, and mount adaptive responses [36]. To assess oxidative stress, several methods are available and are based either on the measurement of the stable peroxidation products (mainly lipid peroxidation products, oxidized proteins and DNA oxidation biomarkers) or the measurements of antioxidants agents.

In our laboratory, a special attention was given to the study of jellyfish toxicity signaling pathways. *P. noctiluca *is the most venomous Mediterranean Scyphozoa [4-6] which represents a danger to sea bathers and causes fishery damages in the Mediterranean Sea. In fact, excluding haemolytic and cytotoxic effects, the cell-damaging action of cnidarians toxins is greatly unknown [7-42].

Hence, our purpose was to investigate the propensity of *P. noctiluca *venom to cause oxidative damage in HCT 116 cells and its associated genotoxic effect. Oxidative stress was monitored by measuring reactive oxygen species (ROS) and malondialdehyde levels, and by evaluating catalase activity. We further looked for DNA fragmentation by means of the Comet assay.

The effect of venom of *P. noctiluca *on the inhibition of cell proliferation was assessed by MTT assay in HCT 116 cells (Figure 1). A dose-dependent decrease in cell viability was clearly observed with increasing concentrations of *P. noctiluca *venom and we determined the IC50 after 24 h which is about 320 μg/ml by MTT assay. Our findings are in accordance with previous data from Mariottini et al. [17] who demonstrated the cytotoxic properties of *P. noctiluca *nematocysts venom that have been experimentally assessed on cultured cells.

There are previous literatures, including the IC50 values of jellyfish venoms from *Chrysaora quinquecirrha *on CCL-13 hepatocyte (IC50 < 1 μg/mL) [43], from *Cyanea capillata *on HepG2 hepatoma cells for 48 h (IC50 = 20.3 μg/mL) [14], and from nematocyst free-tissue of *Rhizostoma pulmo *on V79 lung fibroblast for 3 h (IC50 = 37.6 μg/mL) [44].

Mechanisms whereby the venom of *P. noctiluca *induced the cytotoxic effect are still not understood. Until now, there are no available data regarding the involvement of oxidative stress induced *in vitro *after *pelagia *venom exposure. To evaluate the ability of *pelagia *venom to generate an oxidative stress status, we choose to monitor one of the earliest responses of oxidative stress which is the increase in ROS levels in cells.

Techniques using fluorescent probes have made it possible to estimate ROS in biological samples, allowing their use as an indicator of oxidative stress. So, we have measured ROS production after *Pelagia *venom treatment using DCFH-DA as a fluorescent probe. We have found that increasing concentrations of *P. noctiluca *venom promoted an increase in the production of the fluorescent DCF and consequently a significant ROS generation in a concentration dependent manner (Figure 2).

Levels of early markers of oxidative stress including antioxidant enzymes, may be altered in the presence of lower levels of oxidative stress. To this end, we have monitored the catalase activity. Our results clearly showed that *P. noctiluca *venom enhances catalase activity (Figure 3). The induction of the enzymatic antioxidant defenses after the exposure to *pelagia *venom could be considered as an adaptive response; that is, a compensatory mechanism that enables cells to overcome the damage caused.

To further demonstrate the implication of oxidative stress in venom induced toxicity, we choose to monitor lipid peroxidation. Lipid peroxidation is one of the suggested cytotoxic mechanisms of jellyfish venom. The MDA is an end product of lipid peroxidation, considered as a late biomarker of oxidative stress and cellular damage [45]. It is generally considered as an excellent index of lipid peroxidation [46-48]. We have shown an increase of lipid peroxidation level which seemed related to *pelagia *crude venom concentrations as inferred by the amount of MDA generated, confirming an increase of free radicals production. This fact emphasizes that the oxidative damage is induced by the venom of *P. noctiluca *in cultured HCT 116 cells (Figure 4).

These results are in accordance with a recent study of Marino et al. [42] who demonstrated that venom of *P. noctiluca *induced inflammation which was characterized by lipid peroxidation *in vivo *[42].

Previous studies indicated that jellyfish venoms were able to induce oxidative stress in cells. Indeed, crude venom from the sea anemone *Stichodactyla helianthus *induces peroxidative damage in rat and human erythrocytes [49], besides, Santamaría et al., [50] described that both haemolysis and Lipid peroxidation enriched significant levels in mice erythrocytes as a response to similar concentrations of venom extracts of the Caribbean sea anemone *Bartholomea annulata*. In addition, the haemolytic response resulted sensitive when exposed to antioxidant agents, and we hypothesized that Lipid peroxidation might have been a causative factor of haemolysis. However, our results differ from Marino et al. [22] who pointed out controversially that the hemolysis caused by *P. noctiluca *venom may not be due to free radical formation [22].

It is well known that ROS can induce a number of molecular alterations on cellular components, leading to changes in cell morphology and viability [51]. Overproduction of ROS may induce cell oxidative injury, such as DNA damage [52] leading to genotoxic process [53]. In addition, MDA is a potentially important contributor to DNA damage and mutation [54]. Therefore, we checked if *pelagia *venom was able to induce genotoxicity in HCT 116 cells. For this, we monitored the comet assay (Single-cell gel electrophoresis) which has become a sensitive and rapid method for the detection of DNA damage by strand breaks, open repair sites, crosslinks, and labile sites at the individual cell level. This assay was considered as a predictor of genotoxic activity of chemicals in animal and human cells [55]. Significant increase in DNA damage score showed a concentration-related elevation after treatment with venom of *P. noctiluca*. This induction of DNA damage was associated with *pelagia *venom-induced oxidative stress. In fact, ROS induction and lipid peroxidation seems to be responsible for venom-induced DNA strand breakage.

Protein components of *P. noctiluca *jellyfish venom were separated by using SDS-PAGE, electrophoretical analysis revealed at least 15 protein bands ranging in molecular weights from 120 to 4 kDa (Figure 6). This suggests the complexity of this venom. We have now no evidence, however, whether some of these proteins contribute to the toxic activities of this jellyfish venom, which was observed in the present study.

In conclusion, our study implicates cytotoxicity, oxidative pathways and DNA lesions demonstrated by DNA fragmentation in the venom of *P. noctiluca *toxicities. These toxic activities might be attributed to the protein nature of the venom. Further biochemical investigations are in progress to characterize the different proteinic components of *P. noctiluca *venom from a toxicological study and to clarify their mechanism of action.

## List of Abbreivations

*Pelagia noctiluca*: *P. noctulica*; ATP: Adenosine-5'-triphosphate; ROS: Reactive oxygen species; DCFH-DA: 2', 7'-dichlorofluorescein diacetate; DCF: 2', 7'- dichlorofluorescein; MDA: Malondialdehyde level; LMP: Low melting point agarose; NMP: Normal melting agarose; IC50: the concentration inducing 50% loss of cell viability.

## Competing interests

The authors declare that they have no competing interests.

## Authors' contributions

YA carried out the studies, acquired the data, performed the data analysis, and drafted the manuscript. MB played a major role in the experimental procedures of this study. WZ carried out the statistical analysis. CB revised the manuscript. SA and HB involved in the design and organization of the study and interpreted the results. All authors have read and approved the final manuscript.
